# Global database of leishmaniasis occurrence locations, 1960–2012

**DOI:** 10.1038/sdata.2014.36

**Published:** 2014-09-30

**Authors:** David M Pigott, Nick Golding, Jane P Messina, Katherine E Battle, Kirsten A Duda, Yves Balard, Patrick Bastien, Francine Pratlong, John S Brownstein, Clark C Freifeld, Sumiko R Mekaru, Lawrence C Madoff, Dylan B George, Monica F Myers, Simon I Hay

**Affiliations:** 1 Spatial Ecology and Epidemiology Group, Department of Zoology, University of Oxford, Tinbergen Building, South Parks Road, Oxford OX1 3PS, UK; 2 University Montpellier 1 (UFR Médecine) & CNRS 5290/IRD 224 (UMR ‘MiVEGEC’), Laboratoire de Parasitologie-Mycologie, 34295 Montpellier, France; 3 CHRU de Montpellier, Centre National de Référence des Leishmanioses, Departement de Parasitologie—Mycologie, 34295 Montpellier, France; 4 Children’s Hospital Informatics Program, Boston Children’s Hospital, Boston, Massachusetts, USA; 5 Department of Pediatrics, Harvard Medical School, Boston, Massachusetts 02115, USA; 6 ProMED-mail, International Society for Infectious Diseases, Worcester, Massachusetts 01655, USA; 7 University of Massachusetts Medical School, Worcester, Massachusetts, 01655, USA; 8 Fogarty International Center, National Institutes of Health, Bethesda, Maryland, 20892, USA

## Abstract

The leishmaniases are neglected tropical diseases of significant public health importance. However, information on their global occurrence is disparate and sparse. This database represents an attempt to collate reported leishmaniasis occurrences from 1960 to 2012. Methodology for the collection of data from the literature, abstraction of case locations and data processing procedures are described here. In addition, strain archives and online data resources were accessed. A total of 12,563 spatially and temporally unique occurrences of both cutaneous and visceral leishmaniasis comprise the database, ranging in geographic scale from villages to states. These data can be used for a variety of mapping and spatial analyses covering multiple resolutions.

## Background & Summary

The leishmaniases cause a range of clinical symptoms, from cutaneous lesions to visceral, often fatal, complications^1^. Considered as one of the ‘neglected tropical diseases,’ knowledge of this disease is relatively poor, particularly when compared to conditions with similar burden profiles^2,3^. Indeed, the real burden of this disease remains unknown^4^. Case survey data such as that presented by Alvar *et al.*^5^, whilst informative, is prone to biases associated with reporting and national healthcare provisioning. Alternate methods should be employed in conjunction with such data to compensate for these issues and provide different approaches for estimating disease distribution and burden. An example of mapping the leishmaniases is presented in Pigott *et al.*^6^ where the authors were able to estimate the current global distribution of these diseases by using the database of 12,563 unique records of occurrence presented here and statistical modelling to produce a pixel-based assessment of the likelihood of disease occurrence.

This database comprises occurrence records from 1960 to 2012, and represents a significant expansion in data compared to existing databases^7^. Database creation and management procedures are outlined below, along with a description of the final database structure. Information for accessing the full database is provided here as well in order to (a) allow for replication of the Pigott *et al.*^6^ study, (b) enable the maps to be improved upon as new modelling methods and datasets become available, (c) provide an additional data source for both local-scale and regional mapping studies and (d) provide information for public health organisations on leishmaniasis in specific regions. All data files are available from Dryad (Data Citation 1).

## Methods

The methods described here expand upon those outlined in Pigott *et al.*^6^ by providing more details on data collection and the protocol for positioning of the data. This paper also provides the methodology for standardisation and validation of the occurrence dataset not previously described.

### Data collection

PubMed and Web of Knowledge were searched using the keyword ‘leish*’ for all articles dating up until December 2012. Abstracts for all articles were imported into a bibliographic referencing tool and assessed for relevance, removing articles that did not contain information relating to disease occurrence. From these searches, 4,845 articles were identified and the corresponding texts obtained in full, where possible.

Leishmaniasis occurrence, defined as a report indicating one or more confirmed cases of leishmaniasis, within a specific administrative unit or 5×5 km pixel in a given calendar year, was divided into two categories representing the two main forms of the disease: cutaneous (including cases of localised cutaneous leishmaniasis, diffuse cutaneous leishmaniasis and mucosal leishmaniasis) and visceral leishmaniasis (including cases of post-kala-azar-dermal leishmaniasis (PKDL)). Only autochthonous symptomatic cases were included; wherever possible, cases that were imported were traced back to the original source of infection, otherwise they were excluded. If reports of PKDL also included information on prior VL infection, the latter was recorded; if not, the PKDL case was registered in the database as a VL occurrence. Similarly, only confirmed cases were included. Diagnoses via PCR or parasite cultures, serological tests or microscopic identification were included, as were articles indicating non-specific ‘laboratory diagnosis’.

In addition to this, information was made available from the strain archives of the Centre National de Référence des Leishmanioses (CNR-L) at the Montpellier University Hospital Centre, France. In total, information from 3,465 strains isolated from humans was provided, collected between 1954 and 2013 (in the final database this was restricted to 1960–2012 to be consistent with the literature searches). These strains have been collected by various groups from around the world, cryopreserved in the International Biological Resources Centre of *Leishmania*, Montpellier, France and have subsequently undergone isoenzymatic identification^8,9^. In addition, information from GenBank was extracted by searching for *Leishmania* spp. known to cause disease in humans^10^.

Informal online information sources, including online news articles and ProMED-mail reports^11^, were provided by HealthMap (http://healthmap.org)^12^. The automated system systematically searches various news aggregators, open mailing lists, electronic disease surveillance networks and public health outbreak report feeds. The HealthMap classification system parses out disease and disease-related keywords from the body of these articles as well as classifying the report by one of five classifications—breaking (i.e., information relating to an ongoing outbreak), context (an article supplying background information on the disease, or relating to policy or research articles), warning (articles that suggest an outbreak or unusual case load is likely in the near future), not-disease related (false-positives resulting from the disease term classification system), and old news (referencing previous outbreaks). Duplicate articles, such as identical reports issued by different news services, are identified and processed by the system by aggregating contemporary articles together and checking for similarities. Later human review of the automated assignments ensures data quality and identifies opportunities to improve the automated process. Articles included in our dataset were those categorised as ‘breaking’ with the disease tag ‘leishmaniasis’ and totalled some 109 reports.

### Geo-positioning of data

Each article sourced from the literature search was read and the geographic coordinates of the cases described in each article manually extracted. In many cases, either due to multiple places sharing the same name or to differences in spelling of place names (particularly when translating between different languages), additional contextual information from the article was required for accurate positioning. Each case was reported to the highest degree of spatial resolution available based upon the information provided. This ranged from point locations (indicative of a precise location, such as a village), to areas, termed polygon locations, which correspond approximately to districts (specifically an Admin 2 unit as classified by the Food and Agriculture Organization’s Global Administrative Unit Layers (GAUL) coding^13^), and areas corresponding to states or provinces (the first national subdivision, GAUL Admin 1). For cities or towns, the coordinates of the centre were recorded, unless a specific part of the city (or an explicit latitude and longitude) was described. In the case of district and provincial level data, the approximate centroid of the polygon (obtained using Google Maps; https://maps.google.co.uk) was recorded, to allow for consistent identification in subsequent analyses.

Montpellier’s CNR-L strain archive has geographic tags associated with it (provided by the original survey teams), and those that included at least a sub-national identifier (ranging from villages to provinces) were geo-positioned using Google Maps by the same rules as with the literature-sourced occurrences. GenBank archived material that had associated spatial tags was similarly geopositioned.

HealthMap automatically geopositions the data it parses using a custom-built gazetteer^12^. An algorithm searches for matches between the online article and language-specific terms linked to locations in the geographic reference database, and therefore can identify potential location tags. These matches are then evaluated by a ruleset that attempts to determine the relevance of each location tag (i.e., to distinguish publication relevant tags from outbreak and disease relevant locations) by assessing the number and position of the location keywords within the text. Certain common misleading phrases, such as ‘Brazil nut’ and ‘guinea pig’ are also accounted for in this phase.

### Occurrence standardisation

As the occurrence database was derived from a wide range of disparate sources, we attempted to standardise the occurrence reports both spatially and temporally. Firstly, using the centroid coordinates, all polygon-level data was assigned to a specific admin unit (as defined by GAUL). Point-level data was similarly aligned to a 5×5 km pixel gridded surface of the world. For occurrences that spanned several years, these were disaggregated so that a location that reported cases from 2000 to 2003 (for example) would represent four separate occurrences, one for each year. Occurrence records which were duplicates in both space (either per pixel or per polygon) and time (per calendar year) were removed from the database thus removing the potential for duplicated reports of the same cases. As such, a unique record in the database is defined as the occurrence of one or more cases in a given location in a specific year. Therefore, irrespective of the number of cases or reports that occur within a given polygon or pixel in a given year, they are summarised as one unique occurrence, i.e., an area with 500 cases in a given year is equivalent to an area with 5 cases. By focussing on using unique occurrences, we avoided issues of bias associated with oversampling caused by higher reporting in one location over another, and thus gather a more accurate picture of the global distribution of these diseases, which is particularly important when using presence-absence based modelling techniques.

## Data Records

The database associated with this article (available via Dryad (Data Citation 1) contains the following fields:

1. OCCURRENCE_ID: a unique identifier assigned to each unique occurrence of disease.
2. SOURCE_TYPE: indicating whether sourced from literature, Montpellier CNR-L strain archives, GenBank or HealthMap.
3. LOCATION_TYPE: indicative of point or polygon level data.
4. ADMIN_LEVEL: the administrative level associated with the occurrence. Values are 1 (state or province), 2 (district) and -999 (point).
5. X: the longitudinal coordinate of the point or polygon centroid in decimal degrees (WGS1984 Datum).
6. Y: the latitudinal coordinate of the point or polygon centroid in decimal degrees (WGS1984 Datum).
7. YEAR: the year of occurrence.
8. COUNTRY: the name of the country within which the occurrence lies.
9. DISEASE: indicating whether cutaneous or visceral form of the disease.

## Technical Validation

All occurrences were compared to standardised 5×5 km pixel grids of the world and checked that they fell on land. Those not on land were automatically repositioned to the nearest land pixel. In addition, all occurrences were referenced with the evidence consensus map produced by Pigott *et al.*^6^ This map is a quantitative measure of the consensus of various sources of evidence for the presence or absence of leishmaniasis within that province, scored on a scale from +100 to −100. Importantly, the dataset used to generate the evidence consensus scores included a wider range of data sources (e.g., health organisation statuses and national-level case data) and included data that did not meet our geographic precision inclusion criteria for the occurrence dataset as described below. Occurrences that fell in regions where there was a consensus on disease absence (between −25 and −100) were manually double-checked by the authors to ensure that the latitude and longitude were correct and that the corresponding articles reported a genuine occurrence of leishmaniasis. Finally, any polygon greater in area than one squared degree was removed from the database, as the generalisation of modelling covariates at this scale would be misleading and present a possible source of bias in the model.

**Figure 1:**
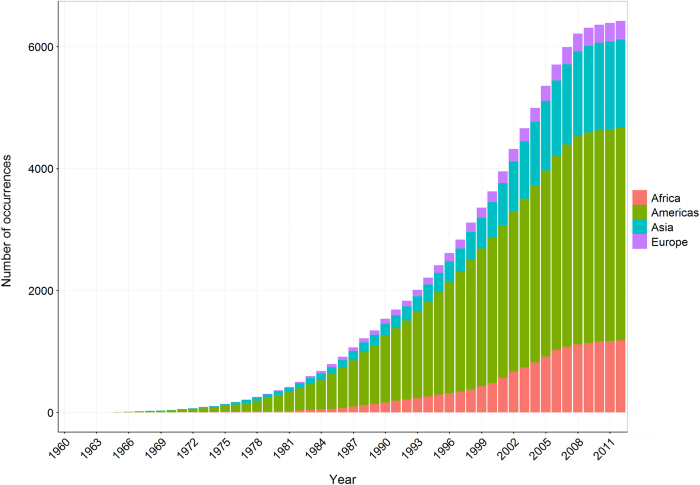
The cumulative number of unique cutaneous leishmaniasis occurrence records per year, from 1960 to 2012, coloured by region (red=Africa, green=Americas, blue=Asia, purple=Europe).

**Figure 2:**
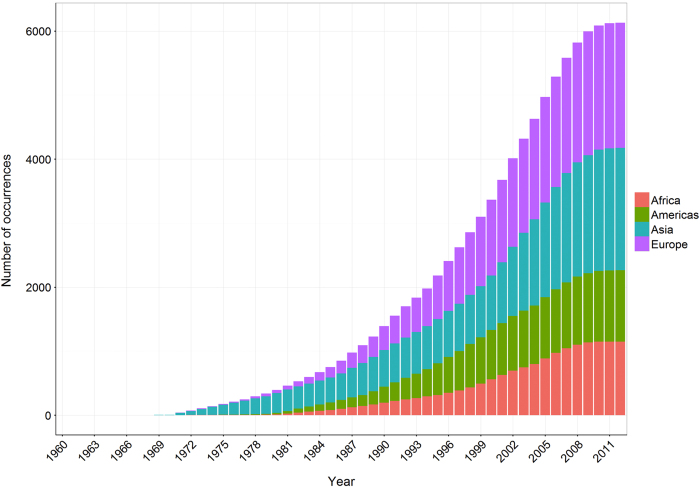
The cumulative number of unique visceral leishmaniasis occurrence records per year, from 1960 to 2012, coloured by region (red=Africa, green=Americas, blue=Asia, purple=Europe).

The final validated database consisted of 12,563 unique occurrences (6,426 for cutaneous leishmaniasis and 6,137 for visceral leishmaniasis) with source locations and occurrence types listed in Table 1. Figs 1 and 2 show the cumulative distributions of unique occurrence records of cutaneous and visceral leishmaniasis over time, subdivided by continent.

## Usage Notes

This dataset can be used to investigate the spatial epidemiology of leishmaniasis at a range of scales, from sub-national studies to regional assessments.

In addition, the framework outlined here can easily be extended to a variety of other diseases, as has already been demonstrated with dengue^14^, and given the potential to automate some of these methods further^15^, the entire process should become increasingly easier and timely. An initial analysis of this dataset was within a niche modelling framework assessing the global distribution of the leishmaniases^6^. The code used to carry out technical validation of the dataset and to generate the predictive risk maps presented in Pigott *et al.*^6^ is freely available as an R software package seegSDM from GitHub (https://github.com/SEEG-Oxford/seegSDM) and is accompanied by a tutorial for its use.

This dataset could be used to assess regional and national variation in disease reporting rates by comparing the density and distribution of occurrence data with existing estimates of case numbers. In areas where there is a lack of regular reporting, occurrences may indicate a potential cryptic burden of disease. Since much of this data is derived independent of centralised healthcare provisioning, it can act as a secondary indicator of disease presence. In addition, the data can be considered in the context of burden estimation, particularly in helping to delineate the spatial limits of leishmaniasis risk.

Despite its comparative ubiquity with respect to other forms of epidemiological data, such as disease prevalence, one of the main limitations of disease occurrence data is the potential for sampling bias^16,17^, where disease occurrence is more likely to be reported from some areas (e.g., those with strong healthcare systems) than others. Consequently, analyses of these data must account for this bias in order to avoid biased conclusions. The importance of this bias is very much dependent on the intended use of the data and a variety of techniques have been developed to assess and accommodate this^17–19^. Pigott *et al.*^6^ mitigated this issue in their predictive mapping study by including an ‘evidence consensus’ map which accounted for reporting rates at a sub-national level. This problem is not only restricted to data from the published scientific literature; data from HealthMap and other internet-based resources are also likely to be influenced by variation in internet usage patterns and online engagement^20^.

## Additional information

**How to cite this article:** Pigott, D. M. *et al.* Global database of leishmaniasis occurrence locations, 1960–2012. *Sci. Data* 1:140036 doi: 10.1038/sdata.2014.36 (2014).

## Supplementary Material



## Figures and Tables

**Table 1:** Origin and spatial resolution of leishmaniasis occurrence data (reproduced from Pigott *et al.*^6^).

	**Point data**	**Admin 1 data**	**Admin 2 data**	**Total**
Origin and resolution of occurrence data				
Cutaneous Leishmaniasis				
*Literature*	3,680	879	1,220	5,779
*CNR-L (Montpellier)*	531	47	31	609
*HealthMap*	31	—	—	31
*GenBank*	6	—	1	7
*Total*	4,248	926	1,252	6,426
				
Visceral Leishmaniasis				
*Literature*	3,050	1,500	1,068	5,618
*CNR-L (Montpellier)*	429	24	29	482
*HealthMap*	32	1	—	33
*GenBank*	3	—	1	4
*Total*	3,514	1,525	1,098	6,137
